# A new soil centipede from South-East Asia with a unique arrangement of ventral glands, and a revised synopsis of Gonibregmatidae (Chilopoda, Geophilomorpha)

**DOI:** 10.3897/zookeys.838.33131

**Published:** 2019-04-15

**Authors:** Binh Thi Thanh Tran, Hoa Thi Xuan Tran, Lucio Bonato

**Affiliations:** 1 Faculty of Biology, Hanoi National University of Education, 136 Xuan Thuy Street, Cau Giay District, Hanoi, Vietnam Hanoi National University of Education Hanoi Vietnam; 2 Pharmacy School, Science Department, University of East Anglia, NR4 7TJ Norwich, Norfolk, UK University of East Anglia Norwich United Kingdom; 3 Dipartimento di Biologia, Università di Padova, via Bassi 58b, I-35131 Padova, Italy Università di Padova Padova Italy

**Keywords:** Chilopoda, Gonibregmatidae, new genus, new species, ventral pore-fields, Vietnam

## Abstract

A new gonibregmatid centipede, *Vinaphilusunicus***gen. n.**, **sp. n.**, is described based on two females from a single location in northern Vietnam. The new genus and species are distinguished mainly by the arrangement of the ventral pore-fields, which is unique among all Chilopoda. A critically revised synopsis of the Gonibregmatidae is also given. In particular, three species are provisionally recognized in *Himatosoma* Pocock, 1891: *H.bidivisum* Silvestri, 1919, *H.porosum* Pocock, 1891 (= *H.typicumtridivisum* Silvestri, 1919, **syn. n.**), and *H.typicum* Pocock, 1891. The genera *Dschangelophilus* Verhoeff, 1937 and *Tweediphilus* Verhoeff, 1937, with their species *D.coloratus* Verhoeff, 1937 and *T.malaccanus* Verhoeff, 1937, are moved to the Gonibregmatidae, whereas *Geoporophilusaporus* Attems, 1930 is moved to the Oryidae as *Orphnaeusaporus* (Attems, 1930), **comb. n.**

## Introduction

Gonibregmatids are frequent members of the centipede communities inhabiting the soils throughout southern Asia (Lewis 1981, Bonato and Zapparoli 2011). They are among the largest and most elongated geophilomorphs, often more than 10 cm long when adults (Bonato 2011). Because of their conspicuous body size, gonibregmatids were among the first centipedes reported from tropical regions (since Newport 1843a, 1843b). Nevertheless, they are still one of the least known centipede groups, even with regards to morphology and species diversity. Moreover, they have been one of the first lineages of geophilomorphs to be distinguished at the family level (Cook 1896), yet researchers are still far from reaching a consensus on the circumscription and diagnosis of Gonibregmatidae (compare e.g. Attems 1929; Bonato 2011; Bonato et al. 2014).

Until now, less than 20 species have been referred to Gonibregmatidae, almost all of them from southern Asia (see Appendix). Most species belong to three morphologically well characterised genera: *Eucratonyx* Pocock, 1899, the best-known genus, with two species; *Gonibregmatus* Newport, 1843, comprising six poorly described and rarely recorded species, of which four are from southern Asia; *Himantosoma* Pocock, 1891, for which up to four nominal taxa have been distinguished and illustrated. A few other incompletely described species of gonibregmatids have been separated in other nominal genera: *Disargus* Cook, 1896, *Geoporophilus* Silvestri, 1919, and *Sogophagus* Chamberlin, 1912.

Recent field sampling in northern Vietnam allowed us to discover a new species that adds significantly to the known morphological diversity of Gonibregmatidae and of Chilopoda as a whole, especially for the unprecedented arrangement of the pores of the sternal glands. The peculiar morphological features of the new species are here described, and its taxonomic position is discussed in the framework of an updated synopsis of the Gonibregmatidae.

## Methods

Specimens were collected by manual search and fixed in 70% ethanol. Morphological examination was performed with a stereoscopic microscope (Leica MZ 125) with magnifications 8–100× and with a biological microscope (Leica DMLB) with magnifications 100–400×, by means of temporary mounts in ethylene glycol and also after dissection of the head and the mouth parts (Pereira 2000). Light photographs were taken with digital cameras applied to the two microscopes (Leica EC3 and DFC420, respectively). Line drawings were drawn from the digital photos. For the morphological description, we followed the standard terminology for centipedes as defined in Bonato et al. (2010).

In order to evaluate the taxonomic position of the new species, we browsed the primary taxonomic and faunistic literature to collate all available morphological information and geographical records for all species either referred or possibly related to Gonibregmatidae. Morphological data were critically revised, and published records were re-interpreted providing modern geographical names.

## Results

Two specimens were found as representatives of a new species of Gonibregmatidae, which deserves to be distinguished in a new genus. It is described below as *Vinaphilusunicus* gen. n. sp. n.

### Vinaphilus gen. n.

Taxon classificationAnimaliaGeophilomorphaGonibregmatidae

http://zoobank.org/6BD441E1-3AB8-47FB-BD63-B6DA3717C02E

#### Diagnosis.

Geophilomorpha differing from all other known genera for the combination of the following characters: both anterior and posterior ends relatively stout; head and forcipular segment quite short; all appendages relatively short; club-like sensilla on multiple distal antennal articles; labrum with broad mid-part separating the lateral pieces; labral margin slightly concave and fringed with elongate, pointed projections; mandible with a single row of many short teeth; telopodite of second maxillae composed of three articles and bearing a claw provided with two marginal rows of thin projections; forcipular tergite wider than long, and as wide as the head; forcipules without denticles; no paratergites; all walking legs with similar claws; sternites with pore-fields, including two paired anterior fields and a single medial posterior field; ultimate leg-bearing segment with entire pleuropretergite; coxal organs opening separately through many pores, most of which aggregated on the meso-ventral side; legs of the ultimate pair much longer than those of the penultimate pair, with two tarsal articles and without claw; female gonopods basally touching, short and non-articulate.

The main diagnostic differences between *Vinaphilus* gen. n. and the other genera of Gonibregmatidae from Asia are summarized in Table 1 and discussed below in Discussion.

**Table 1.** Main differences between *Vinaphilus* gen. n. and all genera of Gonibregmatidae, or possibly belonging to Gonibregmatidae, reported from Asia (see Appendix).

**Nominal genus**	**Type species**	**Labrum**	**Second maxillae**	**Anterior part of trunk**			**Ultimate pair of legs**		
		**Narrow, convex mid-part**	**Filaments on pretarsus**	**Pincer-like claws**	**Paratergites**	**Posterior circular pore-field**	**Aggregated coxal pores**	**Distinctly elongate leg**	**Claw**
*Vinaphilus* gen. n.	*Vinaphilusunicus* sp. n.	–	+	–	–	+	+	+	–
*Disargus* Cook, 1896	*Himantariumstriatum*, by original designation	?	?	?	?	–	–/+	–	+
*Dschangelophilus* Verhoeff, 1937	*Dschangelophiluscoloratus*, by monotypy	–	+	?	–	–	–	–	+
*Eucratonyx* Pocock, 1899	*Himantariummeinerti*, by original designation	–	+	+	–	–	–	+	–
*Geoporophilus* Silvestri, 1919	*Geoporophilusangustus*, by original designation	–	+	?	–	–	–/+	+	–?
*Gonibregmatus* Newport, 1843	*Gonibregmatuscumingii*, by monotypy	+	–	–	+	–	–	+/–	–
*Himantosoma* Pocock, 1891	*Himantosomatypicum*, by original designation	–	+	–	–	–	–/+	–	+
*Luangana* Attems, 1953	*Luanganavarians*, by monotypy	–	+	?	?	–	–	?	–
*Sogophagus* Chamberlin, 1912	*Geophagusserangodes*, by direct substitution	–	+	+	?	–	–	+	–
*Tweediphilus* Verhoeff, 1937	*Tweediphilusmalaccanus*, by monotypy	–	+	?	–	–	–	?	–

#### Type species.

*Vinaphilusunicus* sp. n.

#### Name derivation.

From “Vina”, an alternative name of Vietnam, and the suffix -*philus*, which is frequently used in names of genera of Geophilomorpha. Gender: masculine.

### Vinaphilus unicus sp. n.

Taxon classificationAnimaliaGeophilomorphaGonibregmatidae

http://zoobank.org/E9E2B7EC-DB0F-4F51-ACA8-27C1F78F1144

Figs 1 , 2 , 3

#### Material examined.

*Holotype*: IEBR-Chi 001, ♀, 65 mm long, with 109 leg-bearing segments, with developed gonopods; in ethanol 70%; collected by Anh D. Nguyen, 11–19 September 2016, originally labelled ML01a; originally entire, subsequently divided into four pieces (cephalic capsule with right mandible and part of maxillae; left mandible; left half of the second maxillae; trunk); in the Department of Zoological Museum, Institute of Ecology and Biological Resources, Vietnam Academy of Science and Technology.

*Paratype*: PD-G 9530, ♀, 90 mm long, with 109 leg-bearing segments, with developed gonopods; in ethanol 70%; collected together with the holotype, same date and locality, originally labelled ML01a1; entire; in the Department of Biology, University of Padova.

#### Type locality.

Vietnam: Vinh Phuc province: Ngoc Thanh commune: Me Linh Station for Biodiversity: 21°23'42"N, 105°42'48"E; 150 m a.s.l.; secondary forest.

#### Diagnosis.

A *Vinaphilus* species with the following characters: body length up to > 8 cm; head about as long as wide, lacking transverse suture; antenna about 3 times as long as the head, with intermediate articles about as long as wide; most clypeal setae located in a broad subtriangular medial area, a few other setae close to the mid-point of the anterior margin and in 2 anterolateral groups; intermediate labral projections darker, shorter and closer to each other than lateral projections; forcipular coxosternite > 1.5 times as wide as long, with incomplete chitin-lines; trochanteroprefemur much wider than long; tarsungulum ca 2 times as long as the trochanteroprefemur, with finely serrate internal margin; poison calyx elongated; trunk tergites and sternites wider than long; around 109 pairs of legs, with 2 accessory spines; paired pore-fields of the sternites gradually changing from circular to longitudinally elongated along the trunk, but missing on the first and the prepenultimate leg-bearing segment; medial pore-field of the sternites subcircular, present also on the first and the prepenultimate leg-bearing segment; most of the coxal pores covered by the metasternite of the ultimate leg-bearing segment, a few anterior pores on the lateral side of coxopleuron and a single posterior pore isolated on the ventral side; metasternite of the ultimate leg-bearing segment subtrapezoidal, wider than long; ultimate telopodite ca 2 times as long as the penultimate, with a small terminal spine.

#### Name derivation.

A Latin adjective “*unicus*”, to emphasize the unique arrangement of the ventral glandular pores.

#### Description of holotype.

**Figure 1.**
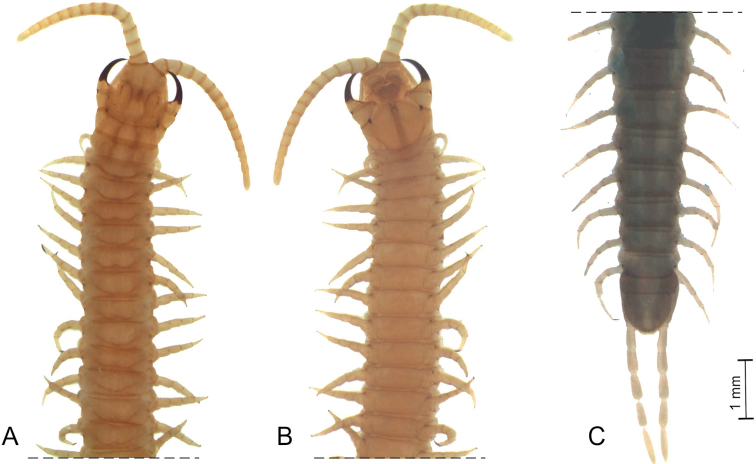
*Vinaphilusunicus* gen. n. sp. n., holotype **A** anterior part of the body, dorsal view **B** anterior part of the body, ventral view **C** posterior part of the body, dorsal view.

*General features* (Fig. 1A–C). Body 65 mm long, depressed, almost uniformly wide along the trunk (ca 1.3 mm) but slightly narrowing posteriorly (penultimate leg-bearing segment 0.8 mm wide). Color (in ethanol) uniformly light yellow, but head and forcipular segment slightly darker.

**Figure 2.**
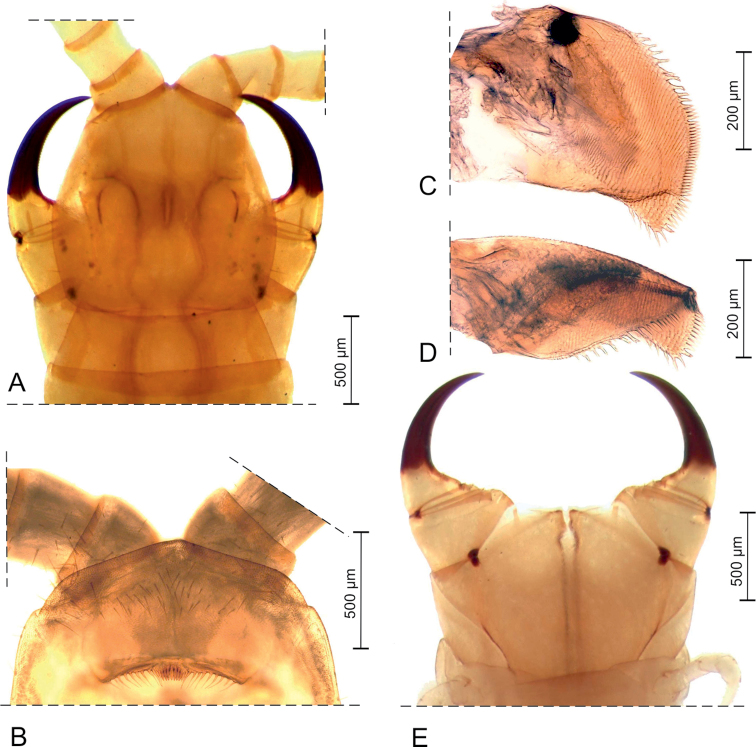
*Vinaphilusunicus* gen. n. sp. n., holotype **A** head and forcipular segment, dorsal view **B** clypeus and labrum, ventral view after removal of forcipules and maxillae **C** left mandible, dorsal view after extraction **D** left mandible, anterior view after extraction **E** forcipular segment, ventral view after removal of head.

*Cephalic capsule* (Fig. 2A, B). Cephalic plate sub-heptagonal, about as long as wide, lateral margins more distinctly converging anteriorly than posteriorly, posterior margin straight; scutes approximately isometric and up to ca 10 μm; transverse suture absent; setae up to ca 100 μm long. Clypeus ca 2.7 times as wide as long, with lateral margins complete; uniformly areolate, with scutes ca 10 μm wide; no obvious clypeal areas; at least 56 setae, all in the anterior half of the clypeus, most of them in a subtriangular intermediate broad area, ca 9 setae in each of 2 anterolateral smaller areas, and a few other apparently broken setae close to the mid-point of the anterior margin of the clypeus. Pleurites uniformly areolate, with 15–17 setae each. Labral margin slightly concave, with a row of 30 projections, including 8 intermediate denticles that are darker, shorter and closer to each other than the lateral projections.

*Antenna* (Fig. 1A, B). 14 articles. Entire antenna ca 2.7 times as long as the head width. Intermediate articles about as long as wide. Article XIV ca 2.1 times as long as wide, ca 1.6 times as long as article XIII, and twice the length of intermediate articles. Setae gradually denser from article I to X on the dorsal side, from article I to V on the ventral side, almost completely missing on the ventral-internal side of articles I–V, however uniformly dense in the remaining distal articles. Setae gradually shorter from article I to about V, up to 100 μm long on article I and < 40 μm long on article XIV. Apical sensilla ca 10 μm long, spear-like, without projections, distinctly narrowing at about the mid-length. Club-like sensilla ca 10 μm long, grouped on the distal parts of the internal sides of articles IX–XIV and on the distal parts of the external sides of articles V–XIV. Three longitudinal rows of 1–5 proprioceptive spine-like sensilla at the bases of the antennal articles, approximately dorsal, ventro-internal and ventro-external; rows reduced to 0–1 spine on antennal articles VI, X and XIV. A few sensilla, similar to the apical ones but slightly darker and shorter, up to 5 μm long, on both dorso-external and ventro-internal position, close to the distal margin of articles II, V, IX and XIII.

*Mandible* (Fig. 2C, D). A single pectinate lamella, with 20–30 elongate teeth. Each tooth about 5 times as long as wide.

**Figure 3.**
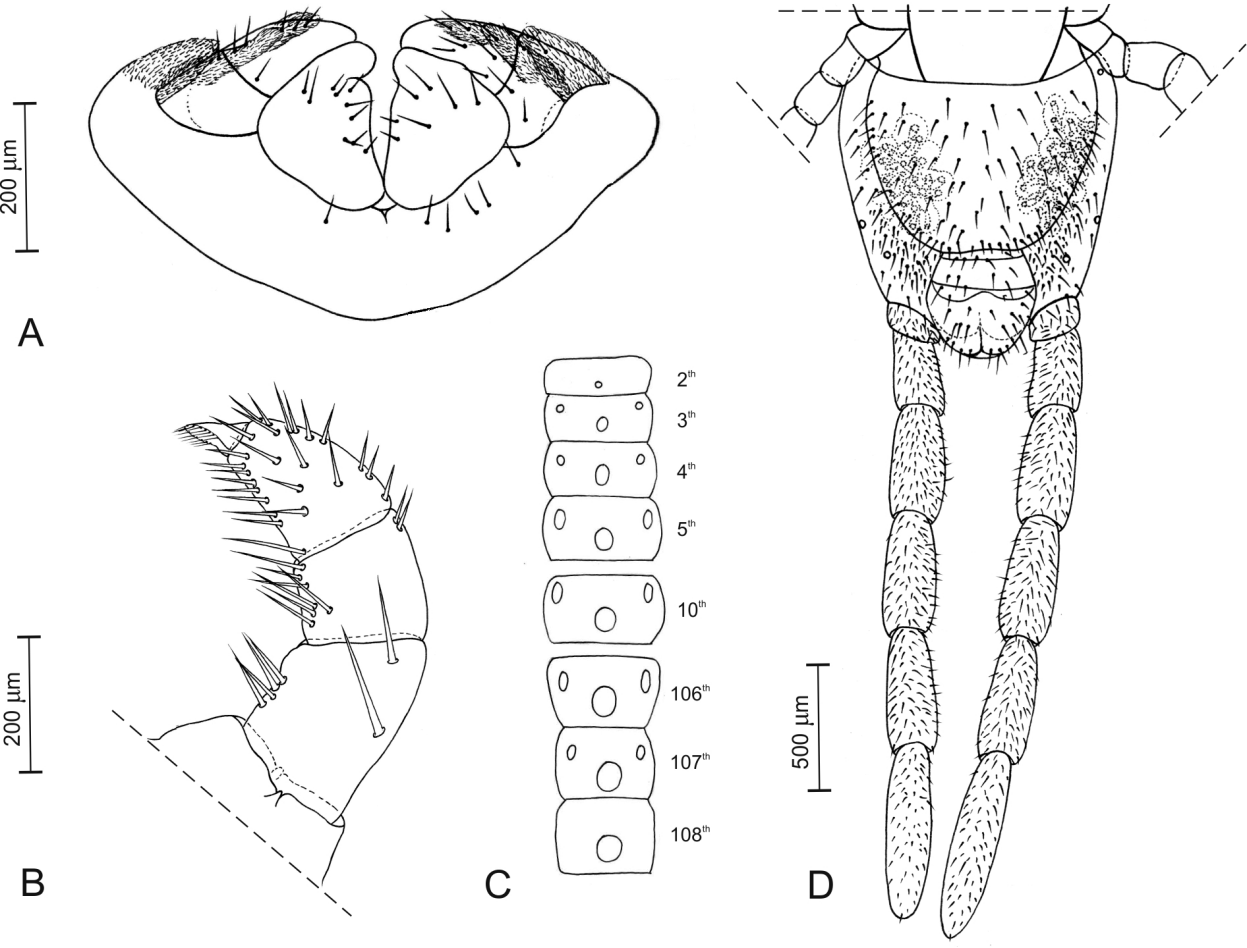
*Vinaphilusunicus* gen. n. sp. n., holotype **A** First maxillae, ventral view after dissection **B** left telopodite of second maxillae, ventral view after dissection **C** sternites of selected leg-bearing segments (indicated by numbers), simplified drawings of pore-fields **D** ultimate leg-bearing segment and postpedal segments, ventral view.

*First maxillae* (Fig. 3A). Coxosternite entire, with setae close to the anterior margin; a pair of lappets, covered with scales. Coxal projection subtriangular, longer than wide, with setae on the ventral side. Telopodite longer than wide, of 2 articles, with setae on the ventral side; a lappet emerging from the basal article and covered with scales.

*Second maxillae* (Fig. 3B). Coxosternite entire, with anterior margin deeply angulated, metameric pores on the central part of each half and about 7 setae near each of the posterior corners. Telopodite of 3 articles, slightly narrowing towards the tip; a number of setae on each article, most of them on the meso-ventral side; pretarsus ca 0.4 times as long as the distal article, slightly bent, narrowing and slightly spoon-shaped at the tip, with 7 filaments along the dorsal edge and 7 filaments along the ventral edge; 4 pore-like sensilla on each pretarsus, 1 on the convex side, 3 on the concave side.

*Forcipular segment* (Fig. 2A, E). Tergite subtrapezoidal, ca 2.8 times as wide as long, with lateral margins strongly converging anteriorly, approximately as wide as the cephalic plate and ca 0.9 times as wide as the following tergite. Exposed part of the coxosternite ca 1.6 times as wide as long; anterior margin with a shallow medial concavity and without denticles; complete coxopleural sutures, entirely ventral, sinuous and diverging anteriorly; chitin-lines incomplete, only visible in the posterior part of the coxosternite. Basal distance between the forcipules ca 0.4–0.5 times of the maximum width of the coxosternite. Trochanteroprefemur ca 1.4 times as wide as long. Intermediate articles distinct. No denticles along the forcipule. Tarsungulum ca 2.8 times as long as wide, ca 2.2 times as long as the trochanteroprefemur; both the external and the internal margins uniformly curved, except for a moderate mesal basal bulge; ungulum not distinctly flattened, internal margin serrated with ca 40 small notches. Elongated poison calyx, ca 5–6 times as long as wide, lodged inside the intermediate forcipular articles.

**Figure 4.**
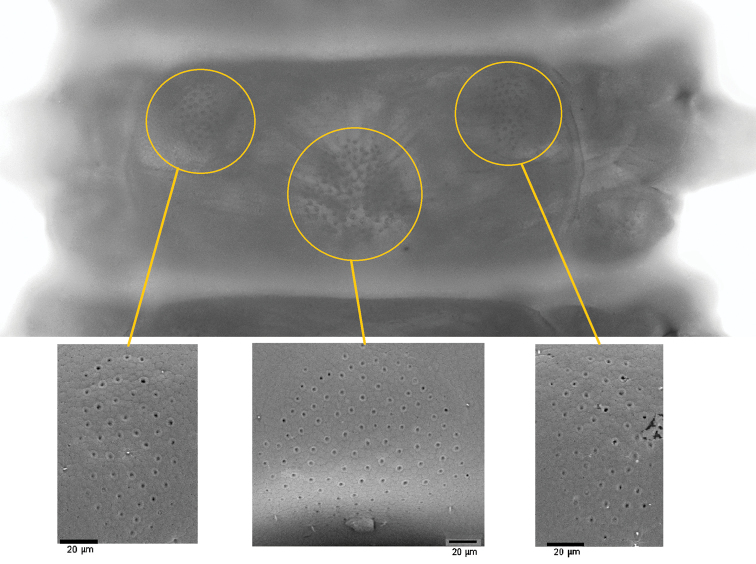
*Vinaphilusunicus* gen. n. sp. n., holotype. Pore-fields of leg-bearing segment 10, ventral view.

*Leg-bearing segments* (Figs 1, 3C, 4). A total of 109 pairs of legs. Metatergite 1 slightly wider than the subsequent one, without pretergite. No paratergites. Walking legs shorter than the width of the trunk; legs of the first pair slightly smaller than the following ones; claws simple, uniformly bent, with 2 accessory spines, the anterior spine reaching at most 20% of the length of the claw, the posterior spine much shorter. Metasternites ca 2 times as wide as long in the anterior part of trunk, up to 1.3 times as wide as long in the posterior part. Ventral glandular pores densely grouped into 3 separate fields on each metasternite, from the second to the prepenultimate leg-bearing segments: 2 paired fields in the anterior part of the metasternite, close to the lateral margins, subcircular and closer to the anterior corners in the most anterior segments longitudinally slightly elongated and closer to mid-length along the remaining trunk; 1 subcircular medial field approximately in the centre of the metasternite in the anterior part of the body, gradually becoming closer to the posterior margin in the most posterior segments. Only the medial field on the first and the penultimate leg-bearing segment.

*Ultimate leg-bearing segment* (Figs 1C, 3D). Pleuropretergite entire, without sulci. Metatergite subtrapezoidal, ca 1.2 times as wide as long, lateral margins convex and converging posteriorly, posterior margin slightly curved. Coxopleuron ca 1.5 times as long as the metasternite. Coxal organs of each coxopleuron opening through about 30 independent pores, mostly clustered and covered by the metasternite, but 2 or 3 pores on the lateral side of the coxopleuron, including 1 pore on the posterior third of the ventral side of the coxopleuron. Metasternite subtrapezoidal, wider than long, anteriorly ca 2.6 times as wide as posteriorly, lateral margins slightly convex and converging backwards; setae denser on 2 broad lateral parts of metasternite, almost absent close to the posterior margin and in a narrower mid-longitudinal stripe. Telopodite approximately 11 times as long as wide, ca 1.8 times as long and ca 1.8 times as wide as the penultimate telopodite; 6 articles; tarsus 2, ca 2.7 times as long as wide and ca 0.7 times as long as tarsus 1; uniformly dense setae, < 45 μm long, on most of the ventral and dorsal sides of the telopodite. Pretarsus absent, a small spine in its place.

*Postpedal segments* (Fig. 3D). Two gonopods, basally touching, subtriangular, without traces of articulation, covered with setae. Anal organs apparently absent.

#### Main differences of paratype.

Entire antenna ca 2.9 times as long as the head width; intermediate articles ca 1.1 times as long as wide; article XIV ca 2.0 times as long as wide and ca 1.5 times as long as article XIII. Exposed part of the forcipular coxosternite ca 1.7 times as wide as long; tarsungulum ca 1.8 times as long as the trochanteroprefemur. Metasternites ca 3 times as wide as long in the anterior part of trunk, up to 1.4 times as wide as long in the posterior part. About 40 coxal pores on each coxopleuron. Telopodite of the ultimate pair of legs approximately 13 times as long as wide, ca 2.3 times as long and ca 1.4 times as wide as the penultimate telopodite.

## Discussion

### Taxonomic position

Considering the most recent comprehensive classification of Geophilomorpha (Bonato 2011) and an even more recent tentative reassessment after a phylogenetic analysis (Bonato et al. 2014), the new species described here is confidently recognisable as belonging to the Geophiloidea. This is especially indicated by the unilamellate shape of the mandibles, which is regarded as a major synapomorphy of the superfamily (Bonato et al. 2014).

More precisely, the structure of the labral sclerites, the shape of the labral marginal projections, the presence of filaments on the second maxillary pretarsi and the structure of the female gonopods of the new species are all plesiomorphic characters within the Geophiloidea and are common to all clades basal to the Geophilidae s.l. (Bonato et al. 2014). However, the family-level taxonomy of the basal geophiloids is still uncertain, especially with regards to the taxonomic circumscription of the traditionally recognized family Gonibregmatidae and the recently established Zelanophilidae (Crabill 1963a, 1963b; Bonato 2011; Bonato et al. 2014: table S2). When considering the elongation of the head and the forcipular apparatus, details of the labrum, and the number of legs, the new species is more similar to the species of *Gonibregmatus* and other gonibregmatids such as *Himantosoma* and *Geoporophilus* than to the species of *Zelanophilus* Chamberlin, 1920 and the related *Tasmanophilus* Chamberlin, 1920. While the above mentioned gonibregmatids have stout head, poorly distinct labral sclerites, short and non-denticulate forcipules and more than 90 pairs of legs (Pocock 1899; Silvestri 1919; Attems 1930), the zelanophilids have slightly elongated head, distinct labral sclerites, usually denticulate forcipules and less than 90 pairs of legs (Archey 1922; Crabill 1963a, 1963b). Worth noting is that the geographical provenance of the new species (north-eastern part of the Indochinese peninsula) is well within the distribution range of the above mentioned gonibregmatids (from the Indian peninsula, through the Indochinese peninsula, the Malay Archipelago and New Guinea, to the Fijian islands; see Appendix), whereas zelanophilids have been reported so far only from Australia, Tasmania, and New Zealand (Bonato 2011, Bonato et al. 2014).

Considering all gonibregmatids so far reported from Asia, including nominal species here recognized for the first time in Gonibregmatidae (see Appendix), we can conclude that the new species differs in at least some major characters that are regarded of high diagnostic value at the species level (Table 2).

**Table 2.** Main differences between *Vinaphilusunicus* gen. n. sp. n. and all other species of Gonibregmatidae reported from Asia (see Appendix). Only reliable sources of morphological information were considered (indicated in the Appendix).

**Species**	**Body**		**Head**	**Labrum**		**Second maxillae**		**Forcipular segment**	**Anterior part of trunk**				**Ultimate leg–bearing segment**				
	**Max length (mm)**	**Pairs of legs**	**Distinct transverse suture**	**Mid-part**	**Marginal projections**	**Telopodite: articles**	**Pretarsus: filaments**	**Complete chitin–lines**	**Paratergites**	**Pincer-like claws**	**Sternite: anterior pore-fields**	**Sternite: posterior pore-fields**	**Anterior extent of coxopleura**	**Coxal pores: arrangement**	**Ultimate/penultimate telopodite length**	**Tarsal articles**	**Claw**
*V.unicus* sp. n.	90	109	–	Wide, concave	+	3	+	–	–	–	2, narrow	1, narrow	Penultimate legs	Almost only meso-ventral, covered	>>1	2	–
‘*B.’ robustus*	58	95	–	Wide, concave	+	?	+	–	?	?	0	0	?	Ventral+lateral+?dorsal	?	2	–
* Di. striatus *	38	69	+	?	?	?	?	?	?	?	1, narrow	1–2, wide	Penultimate legs	Ventral+lateral+dorsal, denser mesally	1	1?	+
* Ds. coloratus *	48	73	?	Wide, concave	+	3	+	?	–	?	1, wide	2, wide	Penultimate legs	Ventral+lateral+dorsal	1	2	+
* E. hamatus *	85	121?–123–125	+	Wide, concave	+	3	+	+	–	+	2, narrow	1, wide	Antepenultimate legs	Ventral+lateral+dorsal	>>1	2	–
* E. meinerti *	130	103–127–129?	–	Wide, concave	+	3	+	+	–	+	2, narrow	2, wide	Penultimate legs	Ventral+lateral+dorsal	>>1	2	–
* Ge. angustus *	55	107	–	Wide, concave	+	3	+	–	–	–	2, narrow	2, narrow	Penultimate legs	Ventral+dorsal, denser mesally	>>1	?	?
* Go. anguinus *	130	115–129	+	Narrow, convex	+	3	–	+	+	–	1, wide	1, wide	Antepenultimate legs	Ventral+lateral+dorsal	1	2	–
* Go. cumingii *	125	161	–	?	?	2?	?	–	+	–	0	0	Antepenultimate legs	Ventral+lateral+dorsal	>>1	2	–
* Go. fijianus *	150	177	+	Convex	?	?	?	?	?	?	?	?	?	?	1	?	?
* Go. insularis *	97	131	–	?	?	?	?	?	+	?	?	?	Antepenultimate legs	Ventral+lateral+dorsal	>>1	2	–
* Go. olivaceus *	110	99–113	–	Narrow, concave	+	3	–	?	+	–	1, wide	1, wide	Antepenultimate legs	Ventral+lateral+dorsal	?	?	–
* Go. plurimipes *	unknown	191	–	Convex	?	?	?	?	?	?	?	?	?	?	>>1	?	?
* H. bidivisum *	45	79	–	?	?	3	+	–	–	–	1, narrow	2, wide	Penultimate legs	Ventral+dorsal, denser mesally	1	2	+
* H. porosum *	42	59–61	–	Concave	+	3	+	–	–	–	1, narrow	1, wide	Penultimate legs	Ventral+dorsal, denser mesally	1	2	+
* H. typicum *	69	57–81	–	Concave	+	3	+	–	–	–	1, narrow	2, wide	Penultimate legs	Ventral+dorsal, denser mesally	1	2	+
* L. varians *	48	55–73	?	Wide, concave	–	3	+?	–	?	?	2	2	Ultimate legs	Ventral+lateral+?dorsal	?	2	–
* S. serangodes *	90	131–135	?	?	–	3	+	+	?	+	0	1, wide	Penultimate legs	Ventral+lateral+dorsal	>>1	2	–
* T. malaccanus *	96	93–107	?	Wide, concave	+	3	+	–	–	?	2, wide	1, wide	Penultimate legs	Ventral+lateral+dorsal	?	2	–

Similarly, with regard to all previously described genera of Asiatic gonibregmatids, including synonyms and other uncertain gonibregmatids (see Appendix), the new species departs from the morphology of all their type species, as well as from the range of variation among the congeneric species, in some major anatomical characters that are commonly considered diagnostic at the genus level (Table 1). The new species does not fit any other genus especially for the unique arrangement of ventral pore-fields (see below) and the unusual arrangement of the coxal pores. Actually, the distribution of the coxal pores somehow resembles the condition found in all species of *Himantosoma* and *Disargus* and in the type species of *Geoporophilus*. In the three latter genera, however, the pores are aggregated on both the meso-ventral and the meso-dorsal sides, and are hardly covered by the adjacent metasternite, which is relatively smaller in comparison with the coxopleura (Pocock 1890; Silvestri 1919).

The new species departs also from all other genera of basal geophiloids inhabiting other continents. In detail, it differs from *Madageophilus* Lawrence, 1960 (a single species from Madagascar, recently referred to Gonibregmatidae; Appendix) in the elongation of the head, the arrangement of the ventral pore-fields and the absence of claws on the ultimate legs (cf. Lawrence 1960). It differs from *Australiophilus* Verhoeff, 1925 (two species from Australia and New Zealand, respectively; variously classified in Geophilidae, Gonibregmatidae or Zelanophilidae; Crabill 1963a; Bonato 2011; Bonato et al. 2014) in the general structure of the labrum, the arrangement of the ventral pore-fields and the coxal pores, and the absence of claws on the ultimate legs (cf. Verhoeff 1925; Crabill 1963a). It differs also from the American genera traditionally separated in the families Eriphantidae (*Eriphantes* Crabill, 1970) and Neogeophilidae (*Neogeophilus* Silvestri, 1918 and *Evallogeophilus* Silvestri, 1918), which have been recently hypothesised to be strictly related to Gonibregmatidae s.s. (Bonato et al. 2014). In particular, the new species differs from *Eriphantes* especially in the general structure of the labrum and the forcipules, the shape of the second maxillae and the arrangement of the ventral pore-fields (cf. Crabill 1970), whereas it differs from the Neogeophilidae in the general body shape and the structure of the first maxillae and the ultimate legs (cf. Silvestri 1918; Crabill 1961).

### Arrangement of sternal glands

As far as known, *Vinaphilusunicus* gen. n. sp. n. is unique among all other gonibregmatids, as well as all centipedes at large, in the arrangement of the ventral pore-fields (Figs 3C, 4).

Ventral glands secreting sticky material are present along the body trunk in most of the geophilomorph centipedes. These glands open through the cuticle of almost all metasternites and often also on the coxae of the adjacent walking legs. The functions of the secretions are largely unknown, but are expected to have a role in deterring predators (Minelli 2011). The arrangement of the glands and the associated pores along the body is conveniently described as a modular longitudinal pattern: all or most glandular pores are grouped in one or more distinct clusters (pore-fields) on the ventral side of each leg-bearing segments. Extent, shape and position of the pore-fields are similar between adjacent leg-bearing segments, with some gradual variation across the longitudinal series of segments. The pattern of pore-fields is highly variable between species and is known to have been highly evolvable in the geophilomorph subclade Adesmata (Turcato et al. 1995).

The particular arrangement of pore-fields in *Vinaphilusunicus* gen. n. sp. (two sublateral paired fields anterior to a single narrow medial field; Fig. 3C) only partially resembles those found in some other gonibregmatids and some geophilids, where distinct anterior and posterior pore-fields may co-occur in single sternites. However, two anterior sublateral paired fields are never associated with a posterior single narrow medial field, rather with either a transverse very broad field (e.g. *Eucratonyx*; Ribaut 1912; Attems 1929) or two paired fields (e.g. *Geoporophilus*; Silvestri 1919). On the other hand, a posterior single narrow medial field is never associated with two anterior sublateral paired fields, rather with another single narrow medial field (*Eriphantes*; Crabill 1970).

## Supplementary Material

XML Treatment for Vinaphilus

XML Treatment for Vinaphilus unicus

## Appendix

### Species of Gonibregmatidae

Species are listed in alphabetic order. Species traditionally separated in the families Neogeophilidae and Eriphantidae are not considered.

***Eucratonyxhamatus* Pocock, 1899**

Original description – Pocock 1899: 67, fig. 2c.

Other sources for morphology – Ribaut 1912: 285, figs 1-19. Attems 1914: 121, figs 14-21. Attems 1929: 342, figs 304-306. Bonato et al. 2011: 23.

Reported specimens – ≥7.

Type locality – “New Britain” [Papua New Guinea].

Geographical records – Indonesia: Ambon Island (Attems 1927); Dabra, in New Guinea (Chamberlin 1939); “Seltutti”, in Aru Islands (Ribaut 1912). Papua New Guinea: Madang (Attems 1914); New Britain (Pocock 1899); Ralum, in New Britain (Attems 1914); New Ireland (Bonato et al. 2011).

***Eucratonyxmeinerti* (Pocock, 1889)**

Original description – Pocock 1889: 289, fig. 1, as *HimantariumMeinerti* [sic].

Other sources for morphology – Pocock 1891: 426, fig. page 427, as *Himantariummeinertii* [sic]. Pocock 1899: 66, fig. 2. Silvestri 1919: 104, fig. 38, as *Eucratonyxmeinertii* [sic]. Bonato et al. 2011: 23, figs 5, 6d, 7i–l. Koch and Edgecombe 2012: 28, 48, figs 20–21. Bonato et al. 2014: figs 2b, 8b.

Reported specimens – >12.

Type locality – “Sullivan Island” [=Lanbi Kyun, Myanmar].

Geographical records – Myanmar: Great Coco Island (Pocock 1891, as *Himantariummeinertii* [sic]); Lanbi Kyun, in Mergui Archipelago (Pocock 1889, as *HimantariumMeinerti* [sic]); Little Coco Island (Silvestri 1919, as *Eucratonyxmeinertii* [sic]); Mawlamyine (Pocock 1891); Palon (Pocock 1891); Reef Island, near Dawei (Pocock 1891). Thailand: Doi Inthanon (Bonato et al. 2011).

***Disargusstriatus* (Pocock, 1890)**

Original description – Pocock 1890: 248, fig. 4, as *Himantariumstriatum*.

Other sources for morphology – none.

Reported specimens – 1.

Type locality – "Madras" [=Chennai, India].

Geographical records – India: Chennai (Pocock 1889).

Taxonomic notes – The species was originally included provisionally in *Himantarium* (Pocock 1890), later in *Himatosoma* by Pocock (1891) and then invariantly in a distinct monotypic genus *Disargus* since Cook (1896).

***Dschangelophiluscoloratus* Verhoeff, 1937**

Original description – Verhoeff 1937: 226, figs 26–31.

Other sources for morphology – none.

Reported specimens – 1.

Type locality – “Telom Valley, Pahang” [Malaysia].

Geographical records – Malaysia: Telom Valley, in Peninsular Malaysia (Verhoeff 1937).

Taxonomic notes – The species was originally classified in Geophilidae in a distinct monotypic genus *Dschangelophilus* Verhoeff, 1937. It was suspected to belong to Zelanophilidae by Bonato et al. (2014). It is here confidently recognised in Gonibregmatidae for the first time, based on the following characters that were described and/or illustrated by Verhoeff (1937): stout head, presence of filaments on the second maxillary pretarsi, short and non-denticulate forcipules, separate female gonopods.

***Geoporophilusangustus* Silvestri, 1919**

Original description – Silvestri 1919: 107, fig. 39.

Other sources for morphology – none.

Reported specimens – 1.

Type locality – “Sumatra: Indragiri” [=Indragiri river, Indonesia].

Geographical records – Indonesia: Indragiri river, in Sumatra (Silvestri 1919).

Taxonomic notes – While *Geoporophilusangustus* was recognized in Gonibregmatidae since Bonato (2011), another species from Sumatra originally classified in the genus *Geoporophilus*, namely *Geoporophilusaporus* Attems, 1930, is here recognised as actually belonging to the family Oryidae and not to Gonibregmatidae. Even though the mandible was described by Attems (1930) as bearing a single pectinate lamella (as found in Gonibregmatidae, but not in Oryidae), many other characters (shape of labrum, structure of first maxillae, shape of ultimate leg-bearing segment, absence of coxal pores) are common to the Oryidae, and especially to the genus *Orphnaeus* Meinert, 1870, and not to Gonibregmatidae. As a consequence, we propose to recognise *Geoporophilusaporus* Attems, 1930 as a provisionally valid species in the genus *Orphnaeus*, with the name *Orphnaeusaporus* (Attems, 1930) comb. n.

***Gonibregmatusanguinus* Pocock, 1899**

Original description – Pocock 1899: 65, fig. 1.

Other sources for morphology – Attems 1914: 119, figs 1–12. Attems, 1926: figs 427, 429, 430, 431, 432. Attems, 1929: 337, figs 295–299. Koch and Edgecombe 2012: 26, 48, figs 18–19. Bonato et al. 2014: fig. 3a.

Reported specimens – ≥11.

Type locality – "New Britain" [Papua New Guinea].

Geographical records – Indonesia: Jayapura, in New Guinea (Attems 1914). Papua New Guinea: Cape Mimias, in New Ireland (Bonato et al. 2014); New Britain (Pocock 1899); Ponam Island (Attems 1927); Ralum, in New Britain (Attems 1914).

***Gonibregmatuscumingii* Newport, 1843**

Original description – Newport 1843a: 181.

Other sources for morphology – Newport 1843b: 502. Newport, 1845: 434, figs 11–14 of pl. 33, fig. 12 of pl. 40. Newport, 1856: 86. Haase 1887: 113, fig. 118.

Reported specimens – 2.

Type locality – “Philippine Islands”.

Geographical records – Philippines: unknown locality (Newport 1843a); Manila (Elera 1895).

***Gonibregmatusfijianus* Chamberlin, 1920**

Original description – Chamberlin 1920: 34.

Other sources for morphology – none.

Reported specimens – 3.

Type localities – “Nadarivatu” [Fiji]; “Vanua Ava” [=Vanuava, Fiji].

Geographical records – Fiji: Nadarivatu, in Viti Levu (Chamberlin 1920); Vanuava, in Kadavu Island (Chamberlin 1920).

***Gonibregmatusinsularis* Pocock, 1894**

Original description – Pocock 1894: 318.

Other sources for morphology – none.

Reported specimens – 1.

Type locality – “Island of Saleyer” [sic] [=Selayar Island, Indonesia].

Geographical records – Indonesia: Selayar Island (Pocock 1894).

***Gonibregmatusolivaceus* Attems, 1930**

Original description – Attems 1930: 170, figs 83–89.

Other sources for morphology – none.

Reported specimens – ≥3.

Type locality – “Swela, Ost-Lombok” [=Suela, Indonesia].

Geographical records – Indonesia: Suela, in Lombok (Attems 1930).

***Gonibregmatusplurimipes* Chamberlin, 1920**

Original description – Chamberlin 1920: 33.

Other sources for morphology – none.

Reported specimens – 1.

Type locality – “Lomati” [Fiji].

Geographical records – Fiji: Lomati, in Kadavu Island (Chamberlin 1920).

***Himantosomabidivisum* Silvestri, 1919**

Original description – Silvestri 1919: 101, fig. 37, as Himantosomatypicumvar.bidivisa.

Other sources for morphology – none.

Reported specimens – 1.

Type locality – “Barkul” [India].

Geographical records – India: Barkul, in Odisha (Silvestri 1919).

Taxonomic notes – This species was originally described as a variety of *Himantosomatypicum* Pocock, 1891 by Silvestri (1919). It was later cited as a subspecies of *H.porosum* Pocock, 1891 by Attems (1938, 1953) and it was recently listed at the species rank by Tran et al. (2013) but without comments. We maintain it provisionally as a separate species for consistency with the common taxonomic practice in Gonibregmatidae, because of obvious morphological differences from both *H.typicum* (shape of ventral pore-fields and arrangement of coxal pores, when controlling for body size) and *H.porosum* (number of legs, shape of ventral pore-fields and arrangement of coxal pores). The name is available according to Article 45.6.4.1 (ICZN 1999). Specimens from Vietnam identified by Attems (1938) as *H.porosumbidivisum* are tentatively assigned to *H.porosum* according to the reported number of legs, as also consistent with the geographical provenance.

***Himantosomaporosum* Pocock, 1891**

Original description – Pocock 1891: 431, fig. page 431.

Other sources for morphology – Silvestri 1895: 719. Attems 1903a: 65, fig. 1. Attems 1903b: 287, figs 7–10. Attems 1914: figs 24–27. Silvestri 1919: 101, fig. 36, as Himantosomatypicumvar.tridivisa.

Reported specimens – ≥8.

Type locality – “Moulmein” [=Mawlamyine, Myanmar].

Geographical records – Indonesia: Cibodas, in Java (Attems 1903a); Sirambi, in Sumatra (Silvestri 1895). Myanmar: Mawlamyine (Pocock 1891). Vietnam: Cap Varella (Attems 1938, as *Himantosomaporosumbidivisum*); Cau Da (Attems 1938); Deo Ca Pass (Attems 1938); Suoi Dau (Attems 1938).

Taxonomic notes – A variety *tridivisa* was originally described for *Himantosomatypicum* Pocock, 1891 by Silvestri (1919) based on a specimen previously identified by the same Silvestri (1895) as *H.porosum*. It was rarely cited as a subspecies of either *H.porosum* (Attems 1938, 1953) or *H.typicum* (Wang 1967). The name is available according to Article 45.6.4.1 (ICZN 1999). It is here synonymized under *H.porosum* [= *H.typicumtridivisum* syn. n.] because the only putative differences with the former species (relative size of coxopleura and number of coxal pores; Silvestri 1919) are explained by expected interindividual variation associated with difference in body size and possibly age.

***Himantosomatypicum* Pocock, 1891**

Original description – Pocock 1891: 429, fig. page 429.

Other sources for morphology – Pocock 1889: 289, fig. 3, as *Himantariumindicum*. Silvestri 1919: 100, fig. 35. Silvestri 1924: 72. Crabill 1969: 38.

Reported specimens – 8.

Type locality – “Moulmein” [=Mawlamyine, Myanmar].

Geographical records – India: Siju Cave, in Assam (Kemp 1924; Silvestri 1924). Myanmar: Kadan Kyun, in Mergui Archipelago (Pocock 1889, as *Himantariumindicum*); Mawlamyine (Pocock 1891); Mergui Archipelago (Silvestri 1919). Thailand: Sakunotayan waterfall (Bonato et al. 2014).

Taxonomic notes – The two syntypes of *Himantosomatypicum* were indicated both from “Moulmein” [=Mawlamyine] by Pocock (1891: 429), whereas a specimen from “King Island” [=Kadan Kyun] had been previously misidentified by Pocock (1889: 289) as *Himantariumindicum* Meinert, 1886 [currently *Polyporogasterindica*] and later referred to *H.typicum* by the same Pocock (1889: 289).

***Sogophagusserangodes* (Attems, 1897)**

Original description – Attems 1897: 476, figs 1–4, as *Geophagusserangodes*.

Other sources for morphology – Attems 1914: figs 13, 22–23. Attems 1929: 343, fig. 307. Crabill 1969: 38.

Reported specimens – 2.

Type locality – “Soah Konorah” [=Soakonora, Indonesia].

Geographical records – Indonesia: Soakonora, in Halmahera (Attems 1897, as *Geophagusserangodes*).

Taxonomic notes – The species was originally classified in Geophilidae in a distinct monotypic genus *Geophagus* Attems, 1897. The latter name was replaced with *Sogophagus* since Chamberlin (1912) because it was a junior homonym of a genus of Coleoptera. The species was suspected to belong to *Eucratonyx* by Bonato et al. (2011).

***Tweediphilusmalaccanus* Verhoeff, 1937**

Original description – Verhoeff 1937: 225, figs 18-25.

Other sources for morphology – Verhoeff 1941: 87. Wang and Tang 1965: 444.

Reported specimens – ≥4.

Type localities – “Gunong Brinchang” [=Gunung Brinchang, Indonesia] and “Telom Valley bei Gunong Siku” [=Telom Valley, near Gunung Siku, Indonesia].

Geographical records – Malaysia: Gunung Brinchang, in Peninsular Malaysia (Verhoeff 1937); Telom Valley, near Gunung Siku, in Peninsular Malaysia (Verhoeff 1937).

Taxonomic notes – The species was originally classified in Geophilidae in a distinct monotypic genus *Tweediphilus* Verhoeff, 1937 and its taxonomic position was never discussed subsequently. It is here confidently recognised in Gonibregmatidae for the first time, based on the following characters that were described and/or illustrated by Verhoeff (1937, 1941): stout head, weakly defined labrum with elongate projections, presence of filaments on the second maxillary pretarsi, short and non-denticulate forcipules, separate female gonopods.

### Species possibly belonging to Gonibregmatidae

Species are indicated with the name currently in use.

***‘Brachygeophilus’ robustus* Attems, 1953**

Original description – Attems 1953: 143; figs 10, 11.

Other sources for morphology – none.

Reported specimens – 1.

Type locality – “Xieng Kuang” [=Xiangkhouang, Laos].

Geographical records – Laos: Xiangkhouang (Attems 1953).

Taxonomic notes – The species was originally classified in the geophilid genus *Brachygeophilus* Brölemann, 1909 and its taxonomic position was not revised after that *Brachygeophilus* was synonymised under *Geophilus* Leach, 1814 (since Foddai et al. 1995). The species was suspected to belong to Gonibregmatidae by Bonato et al. (2016), but its taxonomic position cannot be resolved based on the published information.

***Luanganavarians* Attems, 1953**

Original description – Attems 1953: 143, figs 7–9.

Other sources for morphology – none.

Reported specimens – ≥2.

Type locality – “Luang Prabang” [=Louangphabang, Laos].

Geographical records – Laos: Louangphabang (Attems 1953).

Taxonomic notes – The species was originally classified in Geophilidae in a distinct monotypic genus *Luangana* Attems, 1953, but it was suspected to belong to Gonibregmatidae by Bonato et al. (2016). Its taxonomic position cannot be resolved based on the description and illustrations provided by Attems (1953). Actually, the original description was published posthumously and may be suspected to be actually composite, i.e. based on specimens belonging to different species, as suggested by the very different numbers of legs reported for the type specimens (55 and 73 pairs of legs).

***Madageophiluspauliani* Lawrence, 1960**

Original description – Lawrence 1960: 50, fig. 16.

Other sources for morphology – none.

Reported specimens – 1.

Type locality – “réserve naturelle de l'Andohahela” [=Andohahela National Park, Madagascar].

Geographical records – Madagascar: Andohahela National Park (Lawrence 1960).

Taxonomic notes – The species was originally classified in Geophilidae in a distinct monotypic genus *Madageophilus* Lawrence, 1960, but it was tentatively listed under Gonibregmatidae by Bonato (2011). Its taxonomic position cannot be resolved based on the original description and illustration provided by Lawrence (1960). In particular, the shape of the labrum, of the second maxillary pretarsi and of the pore-fields are suggestive of Gonibregmatidae, however the elongation of the head would be unprecedented for the family.
